# 3D Printing of Metallic Microstructured Mould Using Selective Laser Melting for Injection Moulding of Plastic Microfluidic Devices

**DOI:** 10.3390/mi10090595

**Published:** 2019-09-10

**Authors:** Nan Zhang, Jinghang Liu, Honggang Zhang, Nigel J. Kent, Dermot Diamond, Michael D. Gilchrist

**Affiliations:** 1 Center of Micro/Nano Manufacturing Technology (MNMT-Dublin), School of Mechanical & Materials Engineering, University College Dublin, Dublin 4, Ireland; 2 National Centre for Sensor Research, Dublin City University, Dublin 9, Ireland

**Keywords:** selective laser melting (SLM), 3D printing, micro features, injection moulding

## Abstract

A new method, a 3D printing technique, in particular, selective laser melting (SLM), has been used to fabricate moulds for the injection moulding of thermoplastic microfluidic chips that are suitable for prototyping and early stage scale-up. The micro metallic patterns are printed on to a pre-finished substrate to form a microstructured mould. The dimensional accuracy, surface morphology, bonding strength between the printed patterns and substrate, as well as the microstructure of micro features were all characterized. A microfluidic mould was successfully printed and used directly for injection moulding of cyclic olefin copolymer (COC) microfluidic chips, which were used subsequently to successfully monitor nitrite concentrations in environmental water. The characterization indicated that this new process can be used for fast fabrication of mould tools for injection moulding/hot embossing microfluidic devices. It is faster, more flexible and less expensive than conventional micro-machining processes, although the accuracy and finish are still needed to improve though process optimization and hybrid SLM and machining processes.

## 1. Introduction

Injection moulding is one of the most used technologies for prototyping and the production of plastic microfluidic devices. Mould with micro scale protrusions are important for this process. Currently, precision moulds are generally produced by micro machining or micro electroforming process. Micro machining, such micro milling, is well used in industry for machining stainless steel moulds. However, micro milling requires high precision machine tools and delicate cutters with a diameter as small as 100 µm. Additionally, tool wear during the milling process can influence mould feature precision. Surface cutting tracks and machining burrs can influence the mould finish, resulting in significant labour efforts to remove burrs [1]. Meanwhile, electroforming is one of most used processes for making nickel mould tools using ultraviolet (UV) lithography and/or dry etch, metallization, electroforming process. This process demonstrates high precision but requires expensive masters and takes several weeks to complete the entire process. The thickness uniformity and flatness can be constrained when growing thick deposits [2].

Furthermore, 3D printing has been gradually used for fast prototyping of microfluidics. Some other 3D printing processes, such as stereolithography and inkjet printing, were used as fast methods for the fabrication of microfluidic devices [3,4,5] or for making a master for casting of Polydimethylsiloxane (PDMS) chips [3]. People use 3D printing to make a microfluidic chip due to its fast prototyping process and less effort compared with micro machining. Some work also used 3D printed moulds for fast prototyping of products [6,7,8], e.g., Stratasys and Formlabs. This mould can be used for tens of times for the proof of concept and mould design validation. Additionally, in laboratory, PDMS casting is widely used, due to its ease to use along with UV lithography process or 3D printing inverted channels. However, PDMS is expensive for mass production and proved to be different from thermoplastics, which are used for mass production. There are also physical differences between PDMS and thermoplastics, including cyclic olefin copolymer (COC) or Polymethylmethacrylate (PMMA), in gas permeability, methods of bonding and of surface functionalization, compatibility with different assays (e.g., PDMS absorbs small molecules) and scale-up capability etc [9]. As a result, a fast prototyping thermoplastic chip is required, particularly in cases where scale up production is required.

Metal 3D printing using selective laser sintering/melting has been gradually used in prototyping of micro parts, such as medical stents [10], stainless steel micro electrode arrays [11], micro chemical reactors and fuel cells [12,13,14,15], columns for capillary liquid chromatography [16], and the surface with a roughness of 1.69 µm [17]. The 3D printed metallic flow reactors are available in [18]. More applications can be found in the review paper recently published [19]. This work is trying to use selective laser melting (SLM) to make a fast-metallic mould for prototyping plastic microfluidics. To the authors’ knowledge, this is the first attempt to develop SLM printed microstructured metallic moulds for the injection moulding of microfluidics. This process chain offers the benefit of spanning the gap between using 3D printing for laboratory prototyping of microfluidics and commercial scale production requirements. The present work focuses on studying the precision of feature printing, characterizing the bond strength between the substrate and micro patterns, and the subsequent finishing process. The 3D printed tool is validated for prototyping a microfluidic chip for water quality monitoring. The limitations and future research efforts associated with this work are also discussed.

## 2. Experimental Methods

### 2.1. Printing of Microfluidic Features Using Selective Laser Melting

In the present work, a Mlab cusing laser melting system (Concept laser, Lichtenfels, Germany) was used to print micro scale metallic patterns on a pre-finished substrate. This was a selective laser melting process, whereby a high-energy fiber laser melted fine metal powders locally and the material solidified as it cooled. The stainless-steel CL 20ES powders (316L) (Concept laser, Lichtenfels, Germany) were used for printing the micro patterns. The laser scanning speed was 600 mm/s with a power of 100 W and a layer thickness of 25 µm.

**Figure 1:**
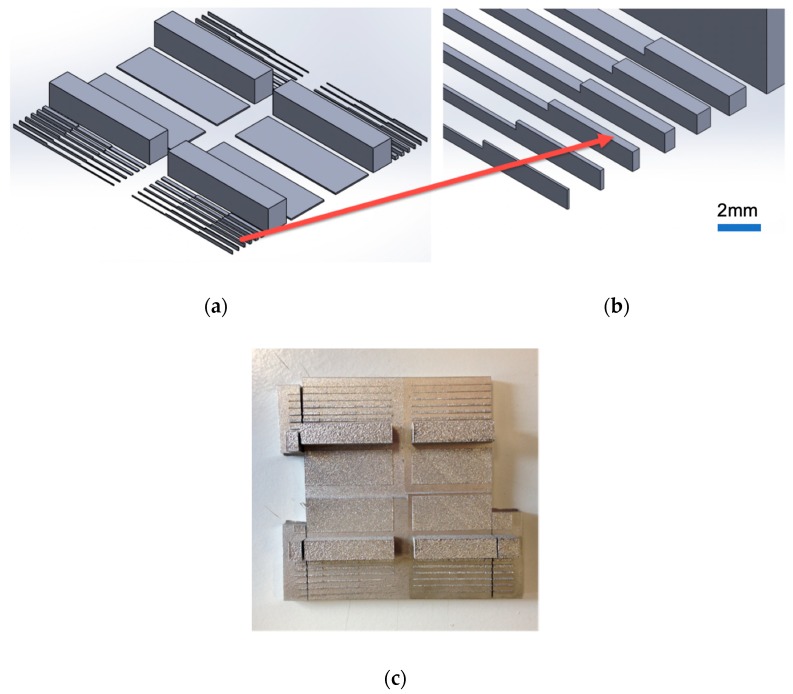
Testing patterns with large ridge and micro ridge arrays: (**a**) four identical testing patterns, (**b**) enlarged view of aspect ratio of rectangular ridges, (**c**) printed patterns on four roughness plates.

A series of generic patterns was designed to test the pattern printing precision and bonding strength with the substrate, as shown in Figure 1a. These rectangular ridges had the widths of 0.1, 0.2, 0.4, 0.6, 0.8, 1 mm and the height 1, 0.6 and 0.3 mm, as shown in Figure 1b. The substrate (17-4PH stainless steel) (Special Steels Ltd., Dublin, Ireland) was pre-machined by die sinking electrical discharge machining (EDM) (Agie Charmilles, Losone, Switzerland) and was pre-located in the powder platform before printing.

**Figure 2:**
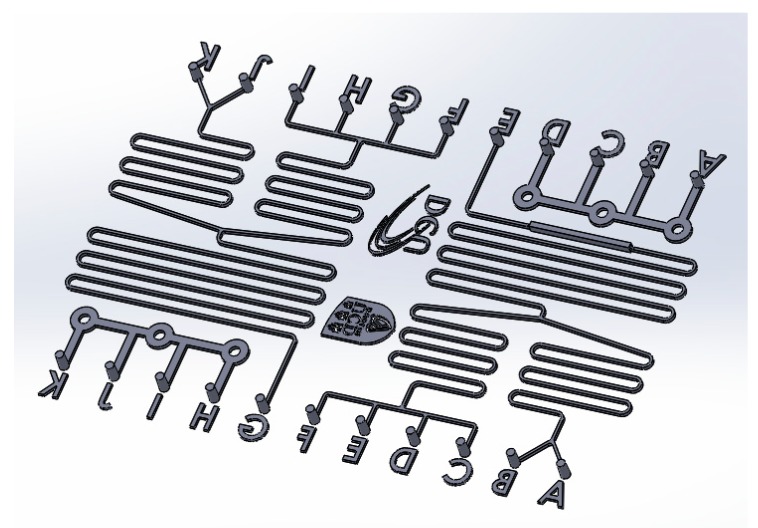
Microfluidic pattern design.

In addition to these test features, a microfluidic pattern was also printed that was designed for water quality monitoring, and which contained inverted channels, marking letters, two logos, and inverted ports, as shown in Figure 2. In terms of the rough surface of SLM, electropolishing was used to finish the tool surface to obtain an acceptable surface roughness for subsequent injection moulding. In the present work, the electropolishing electrolyte was composed of 35% H_2_SO_4_, 45% H_3_PO_4_ and 20% H_2_O, based on work from [20]. 

### 2.2. Bond Strength Testing and Characterization 

A critical risk with this 3D printing manufacturing approach is the possibility of a weak bond strength between the printed metal patterns and the substrate. The substrate surface finish is also important for mould applications. For this reason, electrical discharge machining (EDM) finished surfaces with roughness Ra of 2.5, 1.4, 0.8 and 0.4 µm were tested with four groups of the same printed features (Figure 1c).

**Figure 3:**
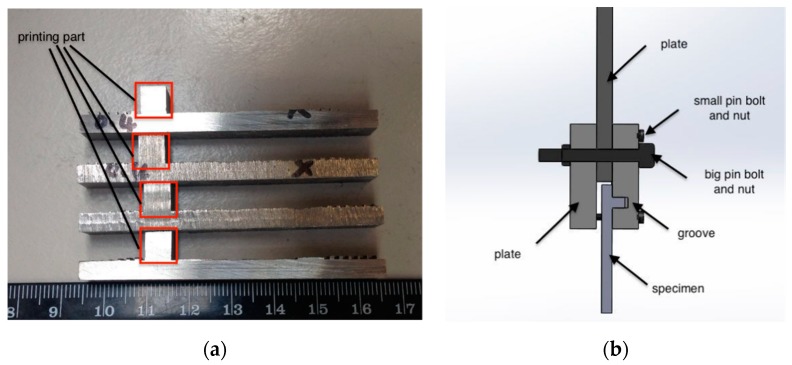
(**a**) Samples for shear testing and (**b**) the corresponding grip.

The scratch tests and lap shear tests were used to evaluate the bonding of the printed patterns. In the preparatory test, a Rockwell indenter was used and scratching was repeated three times across the substrate and printed stainless steel 316L using a nominal load of 5.2 N. Additionally, the printed large ridges in Figure 1c (6 × 7 mm) on each of the four different roughness surfaces were cut into identical samples. The samples with a roughness of 2.5 µm and 0.4 µm were used to carry out shear testing using a standard tensile tester. Figure 3 shows the test samples with one printed block on the substrate. Each sample was gripped by a specially designed holder (Figure 3b) for tensile testing, with shear force being applied to the printed ridge and bond strength being assessed directly.

Generally, failure can happen when it breaks or deforms excessively. As a result, it is important that the level of applied stress never exceeds the ultimate tensile strength or yield strength of the material. It is assumed that the shear force *τ* on a section is uniformly distributed across the whole area. Therefore, the shear stress can be found from [21],

*τ* = *P*/*A* (1)

where *P* is applied force and *A* represents the corresponding area. The design stress is based on yield strength in shear. For many ductile metals, particularly steel, the estimation can be made as follows [21]:

*τ*_d_ = 0.5 *τ*_y_/N (2)

The specification of design shear stress *τ*_d_ depends somewhat on the application. The design stress *τ*_d_ can be based on the yield strength *τ*_y_ or on the ultimate strength *σ*_υ_. Normally, for a dead load on a ductile metal, as in this work, Equation (2) would be used with N = 2, where N is the design factor. The yield strength of stainless steel 316L is 470 MPa. The yield strength in shear is estimated to be 0.58 times the tensile yield strength. Therefore, from Equation (2), the design shear stress of the experimental material can be calculated to be 68 MPa. By comparing the experimental shear stress *τ* against the design shear stress *t*_d_, it can be concluded that if *τ* > *τ*_d_, the SLM printed pattern may be acceptable, but if *τ* < *τ*_d_, the printed pattern may be dangerous.

### 2.3. Microstructure 

The microstructure of printed stainless steel 316L was observed using optical microscopy. The polished sample was etched to expose the microstructure using hydrochloride, glycerine, nitric acid and hydrogen peroxide [22]. The volume percentage of each component is listed as follows: Mass percent concentration of 40% hydrochloric acid: 30–35%; mass percentage concentration of 68% nitric acid: 15–20%; glycerol: 30–35%; mass percent concentration of 30% hydrogen peroxide: 15–20%. The etching was optimized to be 30 s for the substrate, 65 s for a 6 mm-wide feature and 250 s for the smallest feature.

### 2.4. Chip Prototyping Using Injection Molding 

The cyclic olefin copolymer (COC 8007X10) (TOPAS Advanced Polymers GmbH, Frankfurt, Germany) was adopted for injection moulding of the microfluidic chip by using a mould tool insert printed with the pattern of Figure 2. The mould temperature was set at 60 °C and the injection nozzle temperature was 230 °C. The injection speed was set as 100 mm/s and the holding pressure was 50 MPa for 2 s. The designed chip was used as a prototype chip for water quality monitoring.

## 3. Results and Discussion 

### 3.1. Process Development 

**Figure 4:**
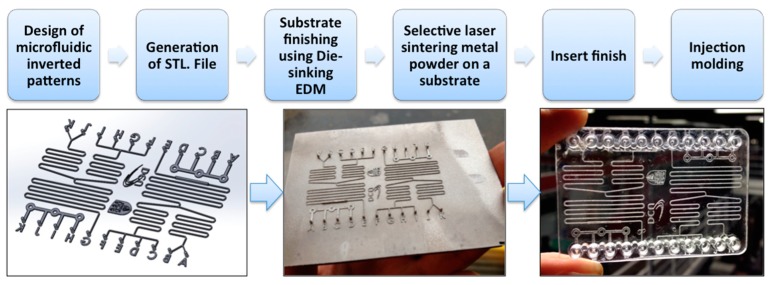
Manufacturing process chain for prototyping plastic devices using selective laser melting (SLM).

The present work developed a new process by directly printing metal micro patterns onto a finished substrate to manufacture a mould tool for the prototyping and manufacturing of polymeric microfluidic devices, as shown in Figure 4. The inverted microfluidic pattern was designed using 3D CAD software (Autodesk, Inc., San Rafael, CA, USA), and this was converted to stereolithographic (STL) format for a laser selective melting machine; the substrate onto which the pattern was printed and was finished using Die-sinking electrical discharge machining or another finishing method. Subsequently, the pattern was directly printed onto the substrate and the substrate was finished as a tool insert to be incorporated into an injection moulding mould. The chip was then fabricated using injection moulding and, finally, the chip was closed with a thermally bonded cover.

The process offers many benefits, namely:

- **High flatness and finish**: Since the substrate can be machined with various finishing processes required by the end applications, the flatness and roughness of the bottom mould insert can be well controlled compared to the insert made by electroforming process and whole selective laser sintering (SLS) printed insert, where the residual stresses can cause part distortion. Compared to micro machining, substrate finished by optical grade die sinking electrical discharge machining shows no cutting marks, requiring no labour cost for subsequent polishing;
- **Cost-effective**: The printing process consumes a minimal amount of material since the inverted microfluidic patterns are only in micrometer scale;
- **Fast prototyping**: The printing process takes less than 20 min, which is notably faster than precision machining and electroforming;
- **Design flexibility**: Any patterns larger than 100 µm are easily printed, even with a freeform geometry;
- **Reusable substrate**: For prototyping, the printed pattern can be removed and the substrate can be reused again;
- **High aspect ratio**: Based on our subsequent work, the aspect ratios of the printed feature can be as high as 5.

### 3.2. Dimensional Accuracy and Surface Finish

**Figure 5:**
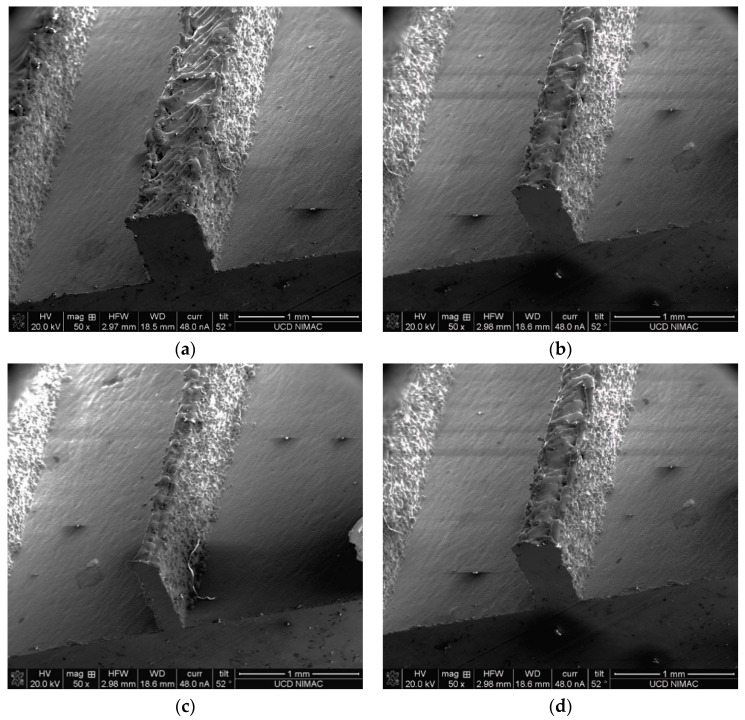
Cross-section of printed features of testing pattern of (**a**) 0.6 mm, (**b**) 0.4 mm, (**c**) 0.2 mm and (**d**) 0.1 mm. Scale bar = 1.0 mm in all cases.

As shown in Figure 5, the high aspect ratios can be achieved. Thus, 100 µm wide patterns can be printed, although patterns larger than 200 µm are easier to define. However, during the SLM process, laser-induced melt splashes are caused by a high capillary instability of the melt, which can form balls on surface of the as-printed part. Additionally, low laser energy also generated highly coarsened balls possessing an interrupted dendritic structure in the surface layer of the as-printed part, which influences surface finish of 3D printed mould tool [23]. This balling phenomenon is related to laser scanning speed, laser power, oxygen content, and powder thickness, and scan interval [23,24], which need to be optimized to establish the best surface finish. Limited by currently accessible SLM systems, the SLM printing parameters were unable to be optimized. Table 1 shows the measured width and height dimensions of the features that are all nominally 600 µm tall. All the feature heights are consistent, ranging from 533–574 µm with dimensional deviation from 26–65 µm. The features of the width 600, 400, 200 and 100 µm show lower width deviation from 2–73 µm.

**Figure 6:**
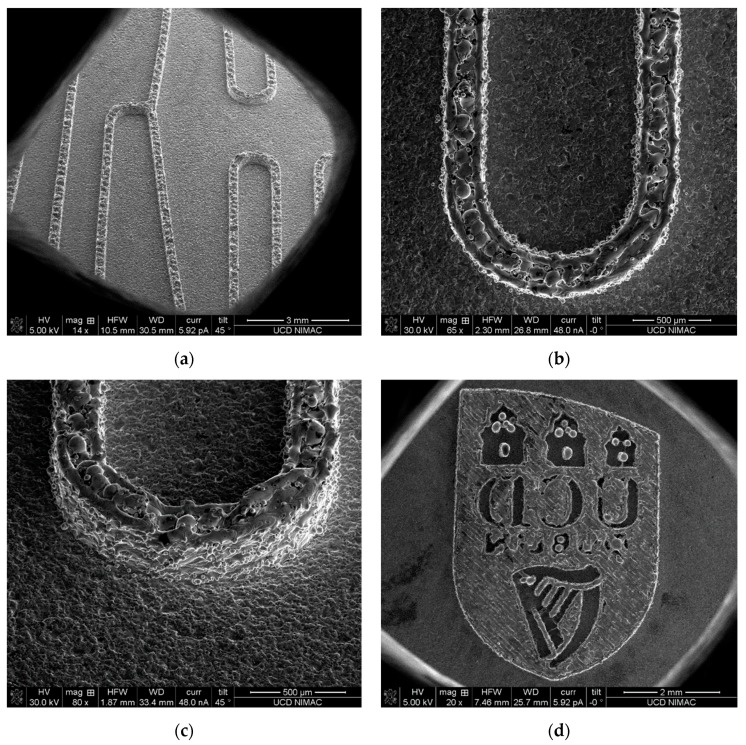
3D printed microfluidic inverted pattern based on SLM: (**a**) inverted channel overview, (**b**) inverted U channel overview, (**c**) tilt view of U channel and (**d**) UCD crest printed from SLM process.

Figure 6 shows the microfluidic features printed on the substrate based on the microfluidic design shown in Figure 2 and Figure 4. The inverted channel width is ~230 µm and height is ~380 µm. Similarly, the top of the inverted channels is not flat, and both sidewalls also show balls and partially melted particles. The ball formation during laser melting at the top of the micro channel makes the channel rougher than the sidewalls, where the ball size is from ~70–100 µm, based on scanning electron microscope (SEM) measurement.

**Figure 7:**
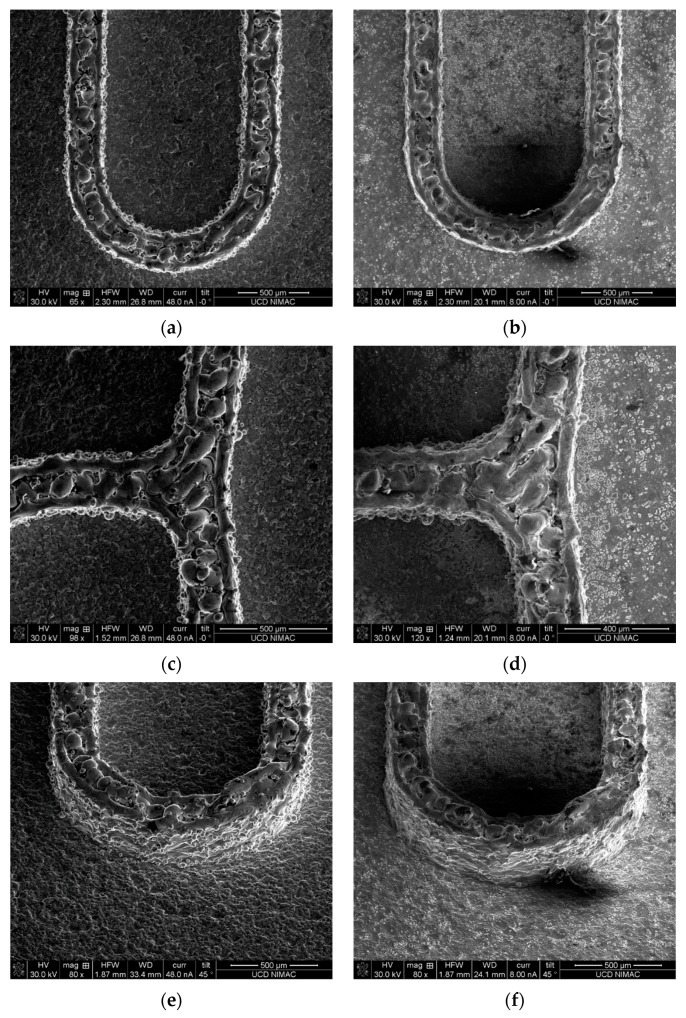

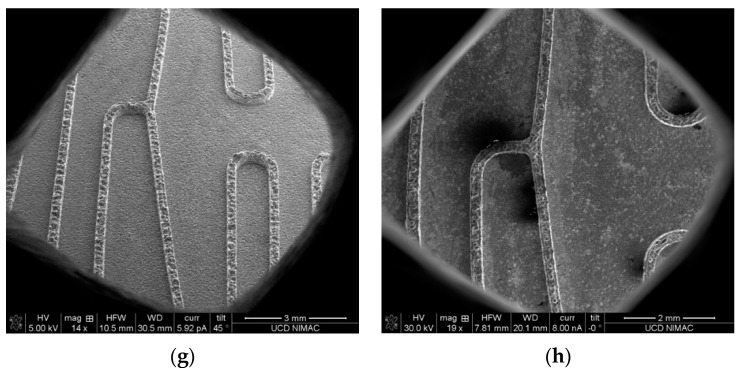
Comparison of tool finish before and after electropolishing: (**a**,**c**,**e**,**g**) are unpolished features and (**b**,**d**,**f**,**h**) are the corresponding polished features (note: scale bar for (**c**,**d**) are 500 and 400 µm, scale bar for (**g**,**h**) are 3 and 2 mm).

**Figure 8:**
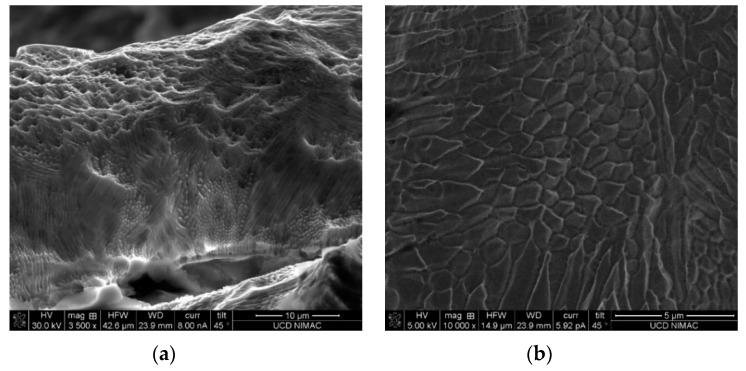
Surface nanostructure on electropolished micro patterns: (**a**) overview of laser melting track after polishing and (**b**) detailed surface submicron features.

Electropolishing was used to finish the tool insert before injection moulding. A current of 0.2 A was used with a gradually increasing polishing time, in order to reduce the significant amount of balls formed on the top and sides of the inverted micro channels. Comparison SEM images, before and after polishing, are shown for the same location of the features in Figure 7. Due to the material removal from the sharp balls, the channel dimension was reduced by ~2.4%. The small un-melted powders were taken away after polishing. Both the channel top and walls exhibit a relatively better surface finish. It is worthwhile to observe that the polished features display dense submicron structures (Figure 8), indicating the effectiveness of polishing. The creation of such submicron structures could provide a new method for large-area sub-micron structuring. However, it still needs to be noted that the balls generated in the laser melting process are so big that the electropolishing process could not provide a surface finish that was comparable to the machined surfaces. This, of course, is an attribute that could be optimized in the SLM process. Additionally, the newly developed hybrid selective laser meting and mechanical machining, such as Sodick OPM250L, can be employed in the implementation of the current strategy, which may eliminate the problem of as-printed rough surfaces [25].

### 3.3. Pattern and Substrate Bonding Strength 

**Figure 9:**
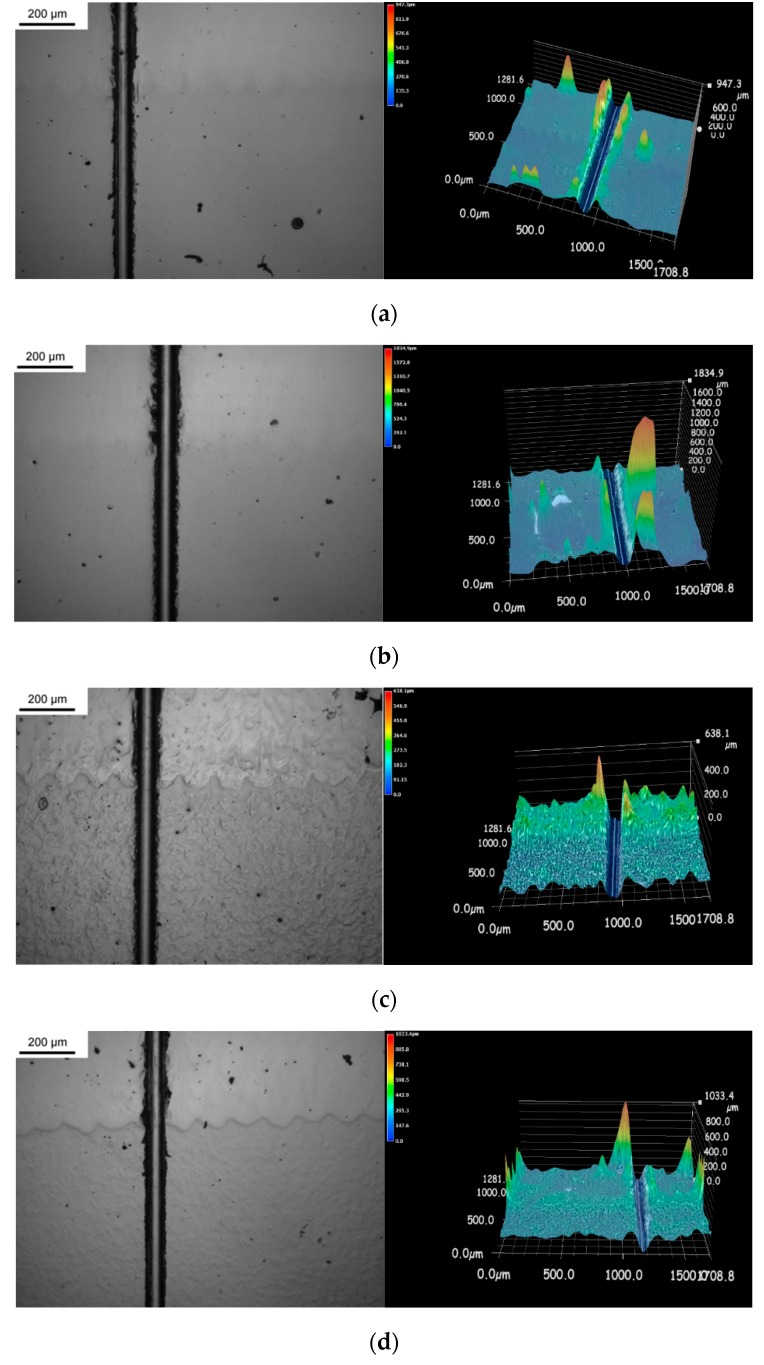
2D top view and 3D view of the scratch across the cross-section of printed metals and substrate: (**a**) Ra 0.4 µm, (**b**) Ra 0.8 µm, (**c**) Ra 1.4 µm and (**d**) Ra 2.5 µm.

The bonding strength of the interface between the printed pattern and substrate was evaluated using the scratch and shear testing. Figure 9 shows the scratch made from printed stainless steel 316L to the substrate. No fracture around the interface over the four roughness substrates was observed. This means that the bonding between the substrate and printed metal is sufficiently strong to sustain the scratching load without any crack or delamination. From the corresponding 3D graphs, it appears that the substrate (17-4PH stainless steel) is harder than the printed stainless steel 316L, with fewer plastic deformation peaks. 

**Figure 10:**
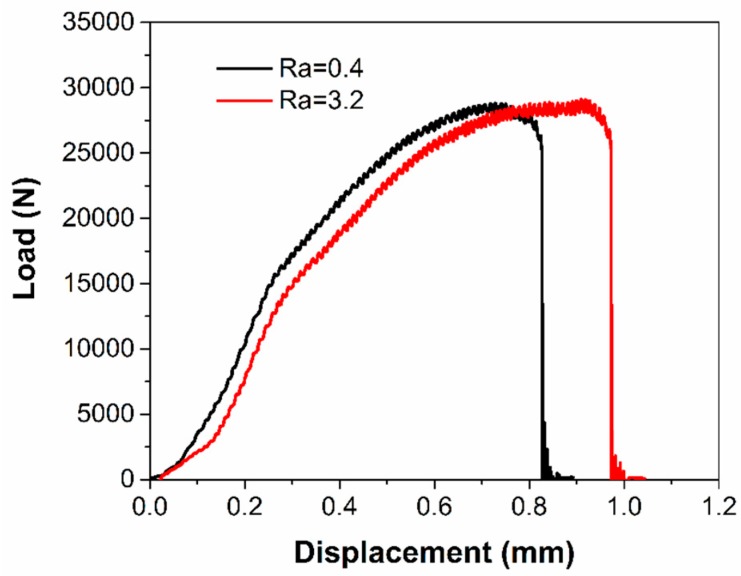
Shear load versus displacement over two roughness substrates.

**Figure 11:**
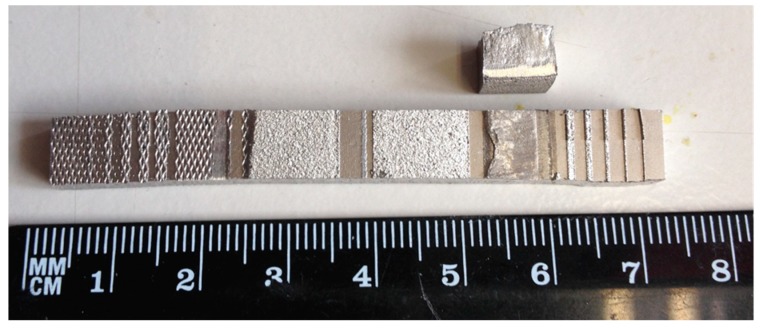
Fracture surface of a printed large ridge and substrate.

**Figure 12:**
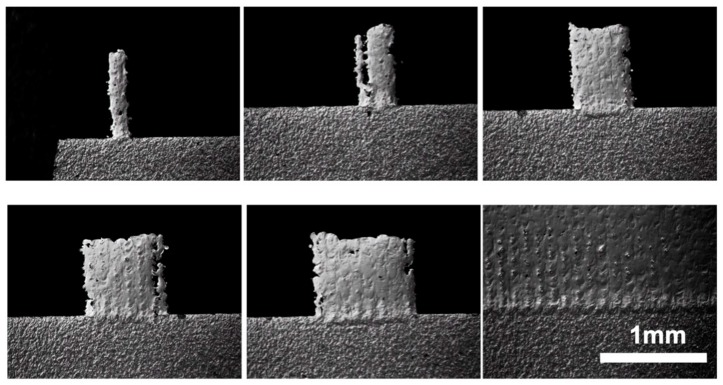
Cross-section of micro features printed by SLM.

Figure 10 displays the load versus displacement (time) at shear testing. The tensile shear strength is obtained directly from the peak load. According to Equation (1), the average tensile shear strength for the Ra = 0.4 µm substrate is 573.4 MPa, while it is 599.8 MPa for the Ra = 2.5 µm substrate. The Ra = 2.5 µm sample has a 4.6% improvement in the shear stress compared to the Ra = 0.4 µm sample. The shear strength is obviously much larger than the design shear stress of 68 MPa. It is also seen in Figure 11 how the fracture surface shows that the crack occurs purely within the printed ridge rather than at the bonding interface. This confirms the sufficient mechanical strength between SLM printed patterns and the substrate. In other words, 3D printing via SLM can provide a strong tool for mould tool applications. As a printed structure, the pores formed by entrapped air may influence the mechanical strength. Figure 12 displays the cross-section of micro features, showing internal pores. Some discontinuous regions of metal are also observed, because of insufficient melting and a lack of diffusion of metal powders. This indicates that the process needs to be optimized. Subsequent heat treatment could be also an effective way to form denser features. 

### 3.4. Microstructures of Micro Features

**Figure 13:**
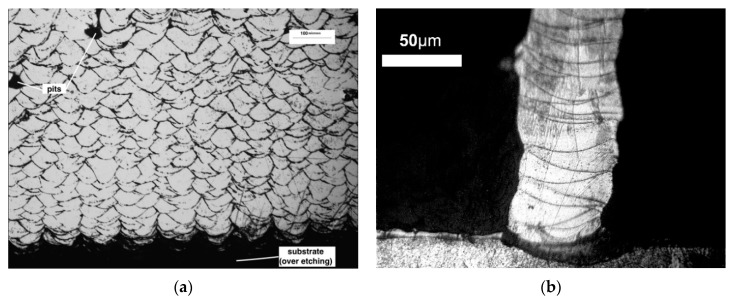
Microstructure of large ridge (**a**) for shear strength test and (**b**) a designed 100 mm feature.

Since the fracture failure happens only within stainless steel 316L, the hardness and microstructure of the features themselves are important to determine the performance of the tools. Figure 13a shows the microstructure of SLM printed stainless steel of 6 mm width for the shearing test. The grain size is approximately 50 µm. These grains are arranged in a fish-scale segregation pattern with a clear indication of the scan layer path from the bottom to the top, which is also the heat transfer direction during laser scanning and solidification, similar to what was found from [26]. Additionally, the voids on the cross-section are also present, due to possible entrapped gas that is insoluble in molten pools of stainless steel 316L, and a possible excessive energy input and unstable process [27]. Figure 13b shows the smallest feature that has a 100 µm nominal width. It can be seen that the single grain layers stack together to form an individual feature. This means that the laser spot size limits the feature size that can ultimately be achieved. Promoting diffusion is important to enhance the mechanical strength of micro features. The hardness of printed stainless-steel micro patterns was measured to be 250HV0.2.

### 3.5. Prototyped Microfluidic Chip Testing 

**Figure 14:**
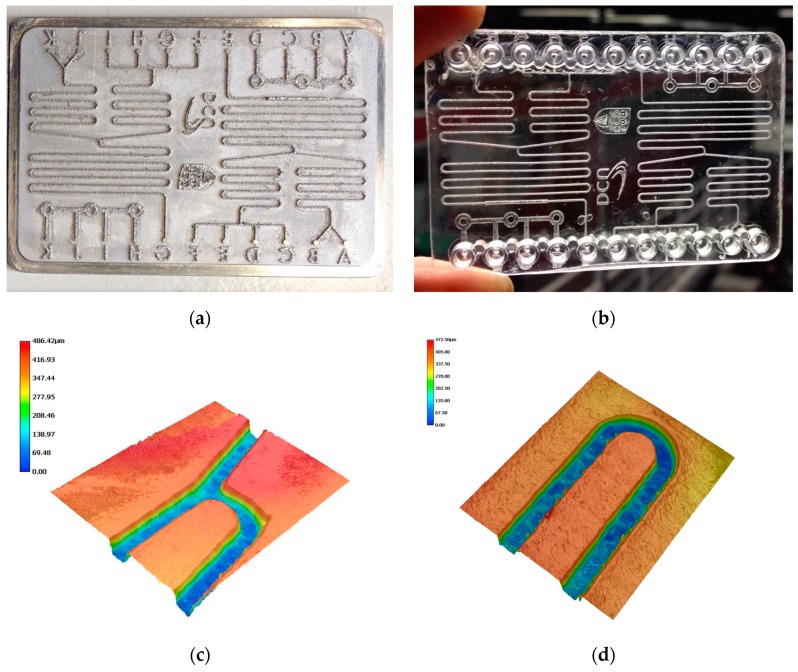
(**a**) SLM printed moulds after polishing, (**b**) injection moulded cyclic olefin copolymer (COC) microfluidic chip, and (**c**) and (**d**) 3D images of plastic chip.

Injection moulding was used to produce microfluidic chips using SLM printed tools, as shown in Figure 14. The chips were then subsequently bonded by thermal diffusion bonding. However, it has to be mentioned that the current printed mould has a rough surface leading to demoulding difficulties. A mould release agent was used to help demoulding of the injection moulded part. Several hundred plastic chips were fabricated without any problem presented from the tool. In Figure 14c,d, the 3D images measured using Keyence VHX-5000 digital microscope (Keyence, Milton Keynes, UK) shows the morphology of the bottom channels of microfluidic chip, where the average roughness is ~96 µm. This value is obviously larger than reported in the literature where average roughness can be as low as ~7 µm [28]. Due to accessibility of the SLM system, the authors could not optimize the printing process. Future work will be carried out with the SLM process optimization and hybrid SLM and the machining process, as discussed in Section 3.7. The injection moulding process can be further optimized based on new mould insert. 

### 3.6. Microfluidic Chip Testing 

**Figure 15:**
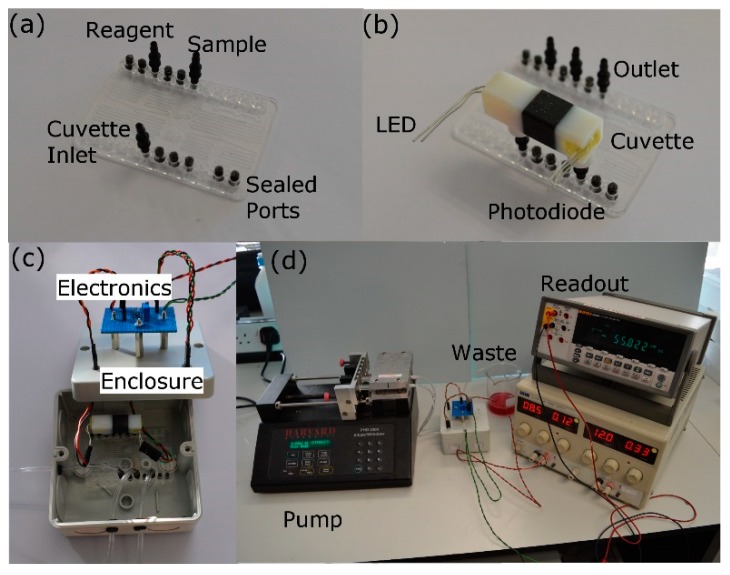
The experimental procedure from chip to system for testing of plastic chip manufactured using the injection moulding process based selective laser melting fabricated tools: (**a**) microfluidic chip with connectors and (**b**) cuvette, and (**c**,**d**) entire enclosure and testing system.

To demonstrate the feasibility of this process, the plastic chip was used for water quality monitoring. In order to test the efficiency of various path lengths, a range of Nitrite concentrations (0–20 µM) were passed through each cuvette. To demonstrate the practical utility of such a chip, one was plumbed in a fashion to enable Nitrite detection, as shown in Figure 15a. Any ports not required for the particular analyte under the test were sealed using standard luer port plugs. In addition to the chip itself, a custom optical cuvette was 3D printed using Polyjet technology, which was used to incorporate light emitting diode (LED) and detector. The principle of operation for Nitrate testing involves mixing a sample containing known values of Nitrate with a reagent to enable proportionate colour changes based on the concentration of Nitrate present in the sample. For this test, the colour change was measured using a combined LED and photodiode. The increased concentrations of Nitrate present in the cuvette would attenuate the incident LED light on the photodiode (Figure 15b). Typically, the chip is placed inside a darkened enclosure to mitigate against photodiode noise caused by ambient light. Furthermore, the additional electronics, such as a constant current source and transimpedance amplifier circuitry, are required to drive the LED and amplify the photodiode signal. The final experimental configuration can be seen in Figure 15d. The configuration shown includes a syringe pump for passing both Nitrate and reagent through the chip, a power supply for the driving electronics and a multimeter to measure the output from the Photodiode.

**Figure 16:**
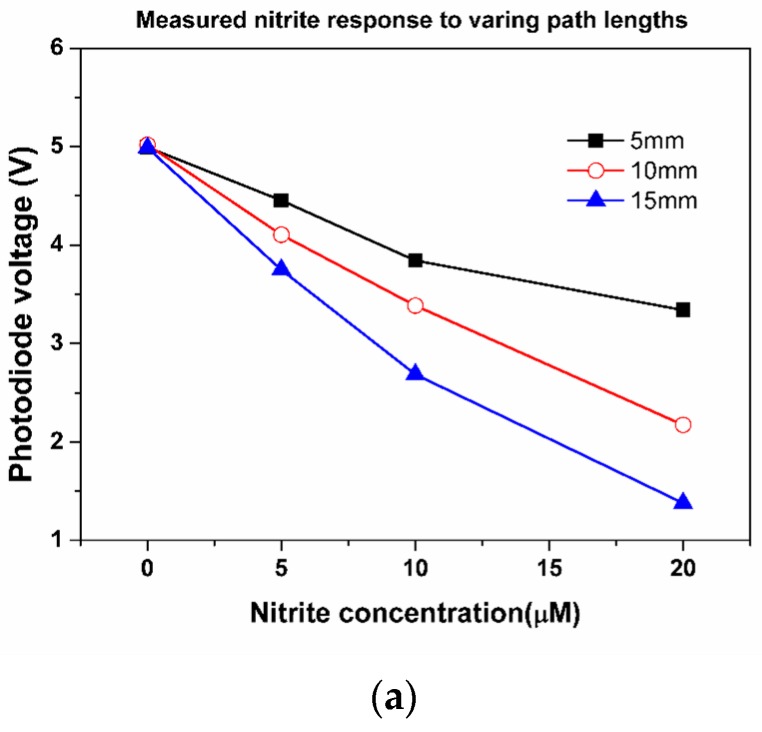
The results from the polymer injection moulded chips. A reduction in photodiode signal (voltage) is observed with the increasing Nitrite signal.

In order to test the range and sensitivity capabilities of the chip, the tests were carried out using three different cuvette path lengths of 5, 10 and 15 mm. For each cuvette length, a drop in the photodiode signal was observed for the increasing Nitrate concentration, as seen in Figure 16. Furthermore, the reduction in measured photodiode signal indicated by photodiode voltage with increasing Nitrite concentration, was more pronounced with the increasing cuvette path length. These findings are entirely consistent with existing literature [29] and demonstrate the utility of the manufacturing process in developing a test platform for nutrient detection.

### 3.7. Discussion and Limitation 

Table 2 compares the properties of these alternative tool fabrication processes based on our experimental trials and experience in ultraviolet lithographie, galvanoformung, abformung (UV LIGA) process. Machining the same micro pattern using a high-speed micromilling machine took at least 6 days to machine the same patterns, i.e., more than two orders of magnitude difference in time alone. This test was done with the same microfluidic pattern design using 5-axis machining centre. For micromachining, the trench size is limited by the milling tool. Depending on the process selected, the size can be from 50 to several hundreds of microns if commercial cutters are used. The micromilling tool was easily damaged and wore when it was used to machine tool the steel. Furthermore, it also can have machining burrs. The LIGA and LIGA like process requires lithography, metallisation and electroforming. It was generally used to make masters for the injection moulding compact disc (CD), digital versatile disc (DVD) and blue rays, with a feature size down to 100 nm. It is also used to make precision micro parts. However, the process takes many steps and takes time to be done. However, the dimensional accuracy is very high, up to 5% of the channel size, and dependent on the precision of lithography and reactive ion etching. SLM printing of micro features on a pre-finished substrate is fast, flexible and sustainable for prototyping and early-stage scale-up for polymeric microfluidics. Since it is only the micro-scale pattern that is printed, only a very small amount of material is used, and printing time is less than 20 min. The printed material and substrate were both stainless-steel, the bonding strength of which was measured up to 600 MPa, which is sufficient to ensure its usage for injection moulding, as has been tested in this work. The substrate was easily reused when a printed pattern was not ideal. These patterns were easily removed by EDM, and new patterns then re-printed.

**Figure 17:**
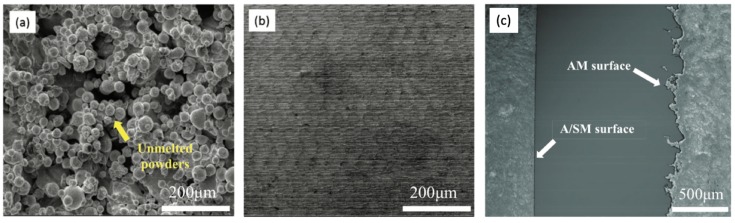
Surface morphology of as-printed parts (**a**), from hybrid SLM and machining process (**b**) and (**c**) [25].

For stainless steel tools, in contrast to precision machining and electroforming, 3D printing based on SLM is a rapid and cost-effective way to prototype tools for polymer microfluidic chips. It is a fast way to validate a particular channel design. However, it must be emphasized that SLM provides a rough surface during laser melting, which is related to laser energy, oxygen concentration, scanning speed, powder size and uniformity etc. The SLM process has to be optimized to obtain an acceptable finish. Additionally, due to the limits of a laser beam size (40 µm), the SLS powder size/uniformity and the layer thickness (25 µm), the resolution of an SLM micro pattern is not as good as can be obtained by precision milling. This means it should only be used in cases where dimension tolerance is larger than 50 µm and the surface roughness does not really matter, such as in the present demonstrated case of a water quality monitoring chip. However, it is worthwhile to notice that with the development of a hybrid SLM and machining process [25], the surface finish and precision can improve for the development of fast mould tools for microfluidics, where the subsequent subtractive milling process can effectively compensate for the surface finish (Figure 17a,b) and dimensional and geometric inaccuracies (Figure 17c) resulting from the powder melting process. The strategy that this work developed and validated can be well implemented for the fast development of mould tools for the injection moulding plastic microfluidics. 

## 4. Conclusions

This present paper develops and discusses a new strategy for the fabrication of micro structured moulds using metal 3D printing via selective laser melting. In this process, a pre-finished substrate was used as a base for printing micro patterns. This printing process only takes ~20 min to make a microstructured mould. It offers many benefits, such as high flatness, low cost, good design flexibility and recyclability of substrates.

Based on the characterization, the bonding strength of a printed pattern to the substrate is ~600 MPa, which is significantly higher than the design shear strength. The interfacial bond is even stronger than printed metal itself, which indicates that the developed process is robust. The pores are also found within the micro patterns and the observed microstructure of the printed patterns indicates that the process can be optimized further. All the feature heights are consistent, ranging from 533–574 µm with a dimensional deviation from 26–65 µm (maximum 11% dimensional error). The features of the width 600, 400, 200 and 100 µm show lower width deviation from 2–73 µm (maximum 12% dimensional error). The replicated channel bottom roughness is ~96 µm, due to the large ball formation at the bottom of the channel (~70–100 µm). Due to the roughness from both the wall and bottom channel, difficulty was experienced for the demoulding of the microfluidic part and a mould release agent had to be used. By using electropolishing, the printed pattern width reduced by 2.4%. The chip was finally fabricated and bonded, and was used successfully in injection moulding for prototyping of a microfluidic chip that could be used for water quality monitoring; nitrite concentrations were shown to be successfully monitored. 

Although the authors have proven that this process is useful for manufacturing microstructured mould tools, the precision and surface finish are still needed to be improved. Future work will focus on the implementation of process optimization and the hybrid process of SLM with machining. By the implementation with such machine tools, the current process can be readily applied for fast fabrication of stainless-steel moulds for injection moulding or hot embossing plastic microfluidic. 

## Figures and Tables

**Table 1:** Dimensions of printed micro feature (unit: µm).

Feature	600	400	200	100
Width	527.1 ± 3.8	349.6 ± 10.8	173.7 ± 6.4	101.9 ± 10.1
Height	549.8 ± 11.7	546.3 ± 15.9	574.8 ± 15.6	566.4 ± 15.3

**Table 2:** Comparison of the fast mould tool prototyping process.

	Minimum Feature Size	Aspect Ratio	Dimensional Accuracy	Young’s Modulus	Roughness (Ra)	Time
Micromilling	50 µm for sunk features	1.5 (features in the range between 50 and 100 µm)	20 µm	180 GPa	0.5–1 µm	6 days
LIGA and LIGA like processes	100 nm	2 (features in the range between 10 and 100 µm)	5 µm	170 GPa	<15 nm	30 days
Selective laser melting	100 µm	6–10 (features width is larger than 100 µm)	50 µm	180 GPa	20~30 µm	~20 min
